# Outcomes of Deceased Donor Kidney Transplantation in the Eurotransplant Senior Program with A Focus on Recipients ≥75 Years

**DOI:** 10.3390/jcm10235633

**Published:** 2021-11-29

**Authors:** Ilias Zompolas, Robert Peters, Lutz Liefeldt, Lukas J. Lehner, Klemens Budde, Bernhard Ralla, Irena Goranova, Andreas Maxeiner, Markus H. Lerchbaumer, Stephan R. Marticorena Garcia, Martin Kanne, Thorsten Schlomm, Matthias R. G. Schulz, Frank Friedersdorff

**Affiliations:** 1 Department of Urology, Charité-Universitätsmedizin Berlin, Corporate Member of Freie Universität Berlin, Humboldt-Universität zu Berlin and Berlin Institute of Health, 10117 Berlin, Germany; ilias.zompolas@charite.de (I.Z.); robert.peters@charite.de (R.P.); bernhard.ralla@charite.de (B.R.); irena.goranova@charite.de (I.G.); andreas.maxeiner@charite.de (A.M.); thorsten.schlomm@charite.de (T.S.); Matthias.schulz2@charite.de (M.R.G.S.); 2 Department of Nephrology and Internal Intensive Care Medicine, Charité-Universitätsmedizin Berlin, Corporate Member of Freie Universität Berlin, Humboldt-Universität zu Berlin and Berlin Institute of Health, 10117 Berlin, Germany; lutz.liefeldt@charite.de (L.L.); lukas.lehner@charite.de (L.J.L.); klemens.budde@charite.de (K.B.); 3 Department of Radiology, Charité-Universitätsmedizin Berlin, Corporate Member of Freie Universität Berlin, Humboldt-Universität zu Berlin and Berlin Institute of Health, 10117 Berlin, Germany; markus.lerchbaumer@charite.de (M.H.L.); stephan.marticorena-garcia@charite.de (S.R.M.G.); 4 Department of Urology, Evangelisches Krankenhaus Königin Elisabeth Herzberge, 10365 Berlin, Germany; m.kanne@keh-berlin.de

**Keywords:** cold ischemia time, delayed graft function, Eurotransplant Senior Program, end-stage renal disease, intensive care unit, kidney transplantation

## Abstract

To evaluate the outcomes of kidney transplantations (KTs) in the Eurotransplant Senior Program (ESP) with a focus on the very old, defined as recipients ≥75 years. This retrospective clinical study included 85 patients, who under the ESP protocol underwent deceased donor kidney transplantation from January 2010 to July 2018 at the Charité–Universitätsmedizin Berlin in Germany. Recipients were divided in three age groups, i.e., Group 65–69, Group 70–74, Group ≥75, and compared. Prognostic risk factors for short and long-term outcomes of kidney transplantations were investigated. Graft survival at 1 and 5 years were respectively 90.7% and 68.0% for group 65–69, 88.9% and 76.2% for Group 70–74, and 100% and 71.4% for Group ≥75. Patient survival at 1 and 5 years were respectively 92.9% and 68.0% for Group 65–69, 85.7% and 61.5% for Group 70–74 and 100% and 62.5% for Group ≥75. Serum creatinine did not significantly differ between the three groups, with the exception of serum creatinine at 1 year. Increased recipient age and prolonged time on dialysis correlated with increased occurrence of postoperative complication. An increase in BMI, pretransplant diabetes mellitus and prolonged time on dialysis correlated with the occurrence of delayed graft function (DGF). History of smoking was identified as an independent risk factor for events of rejection. Increased human leukocyte antigen mismatches (HLA-MM) and prolonged cold ischemia time (CIT) correlated with higher rates of intensive care unit (ICU) treatment. This study supports kidney transplantations for the very old. End-stage renal disease (ESRD) patients ≥75 years of age who underwent kidney transplantation experienced comparable results to their younger counterparts. A comprehensive evaluation of ESRD patients with consideration of prognostic risk factor is the most suitable mean of identifying adequate kidney transplant candidates.

## 1. Introduction

Kidney transplantation is considered the treatment of choice in ESRD, increasing life expectancy and quality of life even for recipients aged ≥65 years [1,2,3,4]. The shortage of renal allograft donors combined with an increased demand from an ever-ageing population has led to the use of expanded criteria donor (ECD) kidneys. ECD kidneys, despite being of lower quality than standard criteria donor (SCD) kidneys, minimize waitlisted time for recipients while providing a survival advantage compared wait-listed dialysis patients [3,4,5,6]. The Eurotransplant Senior Program implemented in 1999, aimed to optimize the allocation of ECD kidneys from deceased donors aged ≥65 to recipients aged ≥65 based on waiting time and blood type compatibility, disregarding HLA matchmaking while minimizing cold ischemia time. Although good results have been reported in recipients aged ≥65 years, only few studies have focused on the potential benefits of kidney transplantations (KTs) in the very old [3,4,5,6,7,8,9]. Studies that evaluated renal allograft recipients over 70 years compared to a waitlisted group or younger counterparts revealed that ≥70-year recipients benefited from the procedure [10,11,12].

However, no scientific research has explicitly assessed the KTs of patients ≥75 years of age thus far. This age group of patients is destined to become clinically more relevant as the number of people aged 75 to 84 years in the EU is projected to increase by 56.1% from 2019 to 2050 [13].

Primary objective of the present study was to evaluate the outcomes of KTs performed under the ESP protocol and to investigate the age limits in recipients. Secondary objective was to identify prognostic factors influencing the short and long-term outcomes of those transplantations with the prospect to improve the pretransplant evaluation.

## 2. Patients and Methods

### 2.1. Study Design

The present retrospective clinical study included 85 patients aged ≥65 years who received a deceased donor kidney transplant from donors ≥65 years allocated through ESP. Recipients were divided into three groups with respect to their age at the time of KT in years as following: Group 65–69, Group 70–74, and Group ≥75. The KTs were conducted by experienced urologic transplant surgeons between January 2010 and July 2018 at the Charité-Universitätsmedizin Berlin. All patients received a renal allograft for the first time and were followed up until death or the end of study (26 May 2020). The immunosuppression protocol after KT was identical for all patients and consisted of tacrolimus, mycophenolate mofetil (MMF) and prednisolone.

This entire analysis was conducted in adherence with the correct scientific research work terms of the Charité Medical University of Berlin, including full anonymization of patient data. All the patients included in the analysis provided written informed consent.

### 2.2. Data Collection and Outcome Measures

Demographic data, medical history, and postoperative follow-up information were extracted through the electronic database Tbase2. Graft characteristics included donor age, number of HLA-mismatches and cold ischemia time (CIT). Specifics of the operations included the side of transplantation, duration of surgery and warm ischemia time (WIT). Serum creatinine levels and glomerular filtration rate (GFR) were used to estimate the renal function of the patients. Short-term outcomes consisted of inpatient stay, occurrence of postoperative complications, Clavien–Dindo classification, DGF, number of dialysis postoperatively, number of days in the ICU, occurrence of rejection and if ICU treatment was required. Long-term outcomes consisted of serum creatinine levels (mg/dl), graft survival, and patient survival at one, three, and five years, death with functioning graft, and patient mortality at last follow-up.

### 2.3. Statistical Analysis

Statistical analysis was conducted using IBM SPSS Statistics, Version 26.0 (Armonk, NY, USA: IBM Corp). Normality of variables was examined with the Kolmogorov-Smirnov test. In order to compare means between groups, the ANOVA test and independent-sample t-test were performed. Fisher’s exact test was carried out to analyze nominal variables. Logistic regression analysis was applied to identify independent risk factors influencing the outcomes using the backward elimination method. Regression models controlled for potential confounders including age of recipient and donor, HLA-MM, body mass index (BMI), diabetes mellitus, hypertension, coronary artery disease, tobacco consumption, time on dialysis, CIT, inpatient stay, DGF, ICU treatment, occurrence of rejection and complications. Survival data was assessed with Cox regression analysis, log-rank, and Kaplan–Meier method with the Group 65–69 set as baseline. *p* < 0.05 was considered significant.

## 3. Results

A total of 85 patients were included in the study with a mean follow-up of 49.72 ± 28.7 months. Demographic data and details regarding the KTs are presented in Table 1 and Table 2. Postoperative course following the KT and long-term outcomes are shown Table 3 and Table 4.

**Figure 1:**
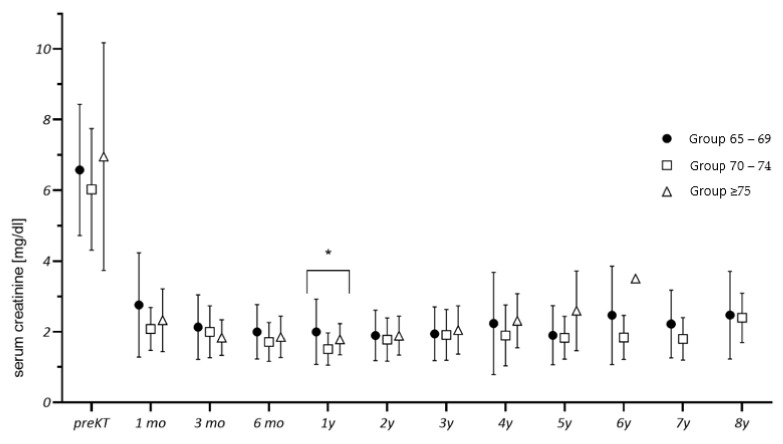
Graft function during follow-up. (* statistically significant difference between the groups).

The logistic regression analysis controlled for potential confounders. The manifestation of postoperative complications correlated with an increase in age of recipient (regression coefficient B = −0.31, odds ratio Exp(B) = 0.74, *p* = 0.049), the occurrence of DGF (B = −3.70, Exp(B) = 0.25, *p* = 0.001), and an increased time on dialysis (B = −0.002, Exp(B) = 0.998, *p* = 0.042). The event of rejection correlated with a history of smoking (B = −1.392, Exp(B) = 0.249, *p* = 0.028) and DGF (B = −2.145, Exp(B) = 0.117, *p* = 0.009). Requirement of ICU treatment correlated with an increase in HLA-MM (B = −2.633, Exp(B) = 0.72, *p* = 0.045) and an increase in cold ischemia time (B = 1.916, Exp(B) = 6.80, *p* = 0.031). Occurrence of DGF correlated with increase in BMI (B = 0.146, Exp(B) = 1.157, *p* = 0.045), longer period on dialysis (B = 0.01, Exp(B) = 1.001, *p* = 0.008), manifestation of perioperative complications (B = 2.423, Exp(B) = 11.28, *p* = 0.001), and diabetes mellitus (B = 1.586, Exp(B) = 4.88, *p* = 0.007). The occurrence of rejection correlated with graft failure (χ^2^ (1, N = 85) = 26.73 *p* < 0.001). There were no significant differences between the three groups after KT regarding serum creatinine, except for creatinine at 1 year (see Figure 1).

**Figure 2:**
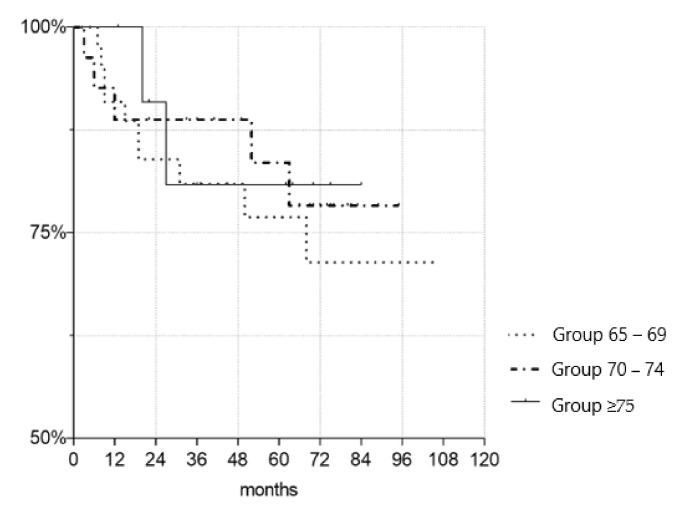
Kaplan–Meier survival plot demonstrating death-censored graft survival. There were no significant differences in the graft survival time between groups (*p* = 0.673 for Gr. 70–74, *p* = 0.814 for Gr. 75+).

**Figure 3:**
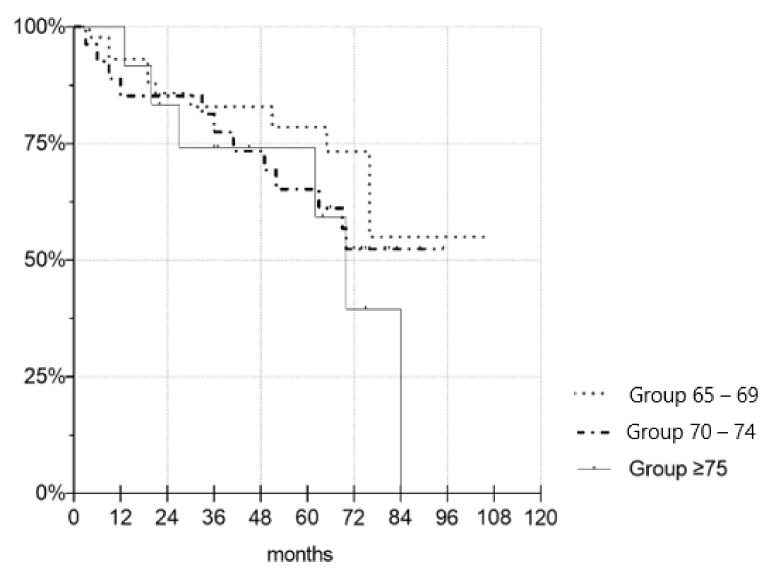
Kaplan–Meier survival plot demonstrating death-censored patient survival. There were no significant differences in the patient survival time between the groups (*p* = 0.149 for Gr. 7074, *p* = 0.438 for Gr. 75+).

Figure 1 depicts the creatinine levels of the three age groups up to last follow-up. Figure 2 and Figure 3 illustrate the death-censored graft and patient survival of the three age groups.

## 4. Discussion

To the best of our knowledge, this is the first study to investigate the clinical outcomes of KT in patients ≥75 years of age. The most important finding is that there were no statistically significant differences in graft and patient survival between the age groups. Recipients aged ≥75 years showed no disadvantages regarding short and long-term outcomes when compared to those aged 65–69 years and 70–74 years. Regarding patient characteristics, pre-existing conditions and ischemia time, no significant differences were established between the three groups except for pre-transplant time on dialysis. Thus, an adequate comparison was possible.

Serum creatinine levels were similar across the three groups up to 5 years of follow-up, suggesting that allograft function was equivalent between the groups. One notable exception was serum creatine levels at 1 year after transplantation, but that difference did not persist.

Overall, recipient and allograft characteristics of this study were similar to those in cohorts examined in recent studies evaluating ESP outcomes [7,8,9]. Quast et al. conducted a single-center retrospective analysis of 217 KTs with a focus on donor age while Badhe et al. focused on prognostic factors for KTs. Graft and patient survival at 1 and 5 years of Quast, Bahde, and Jacobi et al. were comparable to those in groups 70–74 and ≥75 despite recipients in this study being significantly older (7–9) (see Table 5). These results support KT for ESRD patients ≥75 as biological age does not appear to influence the graft or survival of these patients.

Postoperative complications were common with an overall rate of 28.2% and with increased age, DGF, and time on dialysis identified as independent risk factors. Results by Quast and Bahde showed comparable postoperative complication rates at 23.2% and 22.5%, respectively. Jacobi et al., reported 46% of combined peri- and postoperative complications. Inconsistent definition of postoperative complications limits the accuracy of comparisons that can be made. Therefore, this study encourages the adoption of the more objective Clavien–Dindo classification in surgical literature to improve future evaluations.

Independent risk factors for the development of DGF were pre-transplant diabetes, high BMI, longer time on dialysis, and occurrence of perioperative complications. These results are supported by Badhe et al. who identified BMI ≥ 25 kg/m^2^ as a risk factor for DGF and by Parekh et al., who determined pre-transplant diabetes as an independent risk factor in the analysis of 25,523 KTs [7,14]. Previous publications also found that prolonged CIT contributed to a higher incidence of DGF [6,7,11]. However, in this study CIT was kept to a minimum across all groups. This could be the reason that no significant correlation was established between DGF and elongated CIT.

Similarly to previous reports, our analysis identified delayed graft function to meaningfully associated with event of rejection [15,16]. Events of rejection strongly correlated with loss of graft. Preventing such events through adequate selection of transplant candidates and later through well-adjusted immunosuppression is critical.

Nogueira et al. analysis of 997 KT cases found that rejections at 1-year after KT were significantly higher in smokers [17]. This aligns with the results of this study as history of tobacco use correlated with events of rejections. Furthermore, this study established an association of ICU hospitalization with longer CIT and increased HLA-mismatches. It is unclear why HLA-mismatches correlate with higher incidents of ICU hospitalization but not simultaneously with higher incidents of rejection. The current kidney transplant allocation in the ESP with patients over 60 years of age does not take into consideration the HLA mismatches between donor and recipient. A revised model of kidney allocation that considers for HLA compatibility without compromising CIT can prove beneficial in reducing the need for ICU treatment. A reduction in patients requiring ICU is predominantly of value in the ongoing SARS-CoV-2 pandemic, where ICU availability can swiftly become limited.

The proportion of recipients who died with a functioning graft was 65.5%. This is consistent with the findings of Giessing and Boesmueller et al., who described death as the main cause of graft loss [11,18]. The high proportion of patients dying with a functioning graft suggest that even suboptimal allografts can provide adequate function up to the end of the recipient’s life.

The major limitation of this study is its relatively small sample size. Recipients ≥75 years adequate to undergo KT are scarce mainly due to the prolonged waiting time on dialysis. Hence, the assembly of a broader cohort remains challenging. An expansion of the donor pool combined with an increase in kidney donor availability could reduce the waitlisted time and allow for higher rates of transplantation in very old recipients. Additional multi-center studies with bigger cohorts are encouraged to confirm or challenge the results of this study.

## 5. Conclusions

In conclusion, graft and patient survival of recipients ≥75 years was comparable to Group 65–69 and Group 70–74. Therefore, recipients ≥75 years are appropriate candidates for KT and should not be discriminated with respect to their chronological age. An attentive pre-transplant evaluation with consideration of independent risk factors identified as increased time on dialysis, BMI ≥ 25 kg/m^2^, history of smoking, and diabetes mellitus is crucial for transplant outcomes.

## Figures and Tables

**Table 1:** Patient characteristics.

	Groups			
	65–69 years	70–74 years	≥75 years	*p*-Value
*n*	45	28	12	
Gender: male/female	24/16, 53.3%/46.7%	17/11, 60.7%/39.3%	10/2, 83.3%/16.7%	n.s.
Follow-up (months)	46.98 ± 28.6	55.6 ± 30.5	46.25 ± 24.0	n.s.
Age of recipient at time of KT (years)	67.16 ± 1.51	71.86 ± 1.41	77.42 ± 3.30	<0.001
Age of donor at time of KT (years)	71.62 ± 4.38	72.71 ± 5.11	72.92 ± 4.91	n.s.
BMI of recipient (kg/m^2^)	27.4 ± 4.6	27.05 ± 4.05	27.14 ± 3.09	n.s.
HLA-mismatches	3.76 ± 1.28	3.71 ± 1.24	3.67 ± 1.16	n.s.
Primary kidney disease				
Vascular/hypertensive disease	5, 11.1%	11, 39.3%	3, 25.0%	n.s.
Glomerulonephritis	13, 28.9%	7, 25.0%	2, 16.7%	n.s.
Diabetic nephropathy	12, 26.7%	4, 14.3%	4, 33.3%	n.s.
Malignancy	2, 4.4%	0	0	n.s.
Genetic/cystic kidneys disease	8, 17.8%	3, 10.7%	2, 16.7%	n.s.
Infection/reflux	1, 2.2%	0	0	n.s.
Systemic disease	1, 2.2%	0	0	n.s.
Autoimmune	0	0	1, 8.3%	n.s.
Various/unknown	3, 6.7%	3, 10.7%	0	n.s.
Pre-existing conditions				
Arterial hypertension	45, 100%	28, 100%	12, 100%	n.s.
Diabetes mellitus	21, 46.7%	11, 39.3%	5, 41.7%	n.s.
Coronary artery disease	16, 35.6%	14, 50%	4, 33.3%	n.s.
Tobacco consumption	17, 37.8%	5, 17.9%	2, 16.7%	n.s.
Previous operations in abdominal region	20, 44.4%	6, 21.4%	9, 75%	n.s.
Dialysis				
Hemodialysis	37, 82.2%	27, 96.4%	11, 91.7%	n.s.
Peritoneal dialysis	8, 17.8%	1, 3.6%	1, 8.3%	n.s.
Time on dialysis (days)	1950 ± 840	1487 ± 461	1418 ± 527	0.008

All values with *n*, percent or mean and standard deviation. n.s = not significant.

**Table 2:** Surgery details.

	Groups			
	65–69 years	70–74 years	≥75 years	*p*-Value
Side of transplantation: fossa iliaca dextra/sinistra	26/19, 57.8%/42.2%	16/12, 57.1%/42.9%	6/6, 50%/50%	n.s.
Operation time (minutes)	203 ± 52.7	202 ± 46.9	235 ± 33.7	n.s.
Cold ischemia time (hours)	10.05 ± 3.78	9.46 ± 3.29	9.11 ± 2.96	n.s.
Warm ischemia time (minutes)	48.1 ± 10.7	52.6 ± 14.5	49.8 ± 10.0	n.s.

All values with *n*, percent, or mean and standard deviation; n.s = not significant.

**Table 3:** Postoperative course.

	Groups			
	65–69 years	70–74 years	≥75 years	*p*-Value
Inpatient stay (days)	22.1 ± 13.0	21.5 ± 15.6	19.3 ± 10.2	n.s.
Occurrence of postoperative complications	12, 26.7%	10, 35.7%	2, 16.7%	n.s.
Clavien–Dindo classification				
Clavien–Dindo 1	7, 15.6%	8, 28.6%	n.a.	
Clavien–Dindo 2	n.a.	n.a.	n.a.	
Clavien–Dindo 3	5, 11.1%	2, 7.1%	2, 16.7%	
Delayed graft function	27, 60%	14, 50%	5, 41.7%	n.s.
Number of dialysis postoperatively	6.44 ± 7.63	2.57 ± 2.10	4.80 ± 3.96	n.s.
ICU required	12, 26.7%	6, 21.4%	3, 25.0%	n.s.
ICU duration (days)	1.58 ± 0.9	4 ± 3.58	3.0 ± 1.73	n.s.
Occurrence of rejection	10, 22.2%	5, 17.9%	1, 8.3%	n.s.
Cause of rejection				
Acute rejection	4, 8.9%	2, 7.1%	1, 8.3%	
Chronic rejection	4, 8.9%	2, 7.1%	0	
Vascular complications	1, 2.2%	0	0	
Tumor	1, 2.2%	0	0	
Infection	0	1, 3.6%	0	

All values with *n*, percent or mean and standard deviation. n.a. = not applicable; n.s = not significant.

**Table 4:** Long-term outcomes.

	Groups			
	65–69 years	70–74 years	≥75 years	*p*-Value
Creatinine levels (mg/dL)				
Preoperatively	6.57 ± 1.86	6.03 ± 1.72	6.95 ± 3.32	n.s.
1-year	1.99 ± 0.93	1.51 ± 0.46	1.79 ± 0.44	0.046
3-year	1.93 ± 0.76	1.91 ± 0.72	2.05 ± 0.68	n.s.
5-year	1.89 ± 0.83	1.82 ± 0.61	2.19 ± 1.13	n.s.
Graft survival				
1-year	90.7%	88.9%	100%	n.s.
3-year	79.4%	80.0%	80.0%	n.s.
5-year	68.0%	76.2%	71.4%	n.s.
Patient survival				
1-year	92.9%	85.7%	100%	n.s.
3-year	79.4%	77.8%	72.7%	n.s.
5-year	68.0%	61.5%	62.5%	n.s.
Patient mortality at last follow-up	11, 24%	12, 42.9%	6, 50%	
Of these: death with functioning graft	8, 72.7% *	7, 58.3% *	4, 66.7% *	
Cause of death				
Cardiovascular	2/4.4%	0	0	
Graft failure	1/2.2%	1/3.6%	0	
Infection/sepsis	3/6.7%	8/28.6%	2/16.7%	
Malignancy	4/8.9%	1/3.6%	2/16.7%	
Traumatic	1/2.2%	2/7.1%	2/16.7%	

All values with *n* (percent) or mean and (standard deviation, SD). *p* < 0.05, * Percentage is the result of *n* of patients with functioning graft divided by *n* of deceased patients at last follow-up; n.s = not significant.

**Table 5:** Comparison of death-censored graft and patient survival in the Eurotransplant Senior Program.

	Quast(9)	Bahde(7)	Jacobi(8)	Our Results		
	*n* = 217	*n* = 89	*n* = 89	Group 65–69	Group 70–74	Group ≥75
Age of recipients at KT	68.1 ± 3.8	72.2 (70–77)	68.2 ± 3.2	67.16 ± 1.51	71.86 ± 1.41	77.42 ± 3.30
Graft survival						
1-year	76.4%	n.a.	87%	90.7%	88.9%	100%
5-year	57.3%	77%	63%	68.0%	76.2%	71.4%
Patient survival						
1-year	88.2%	n.a	87%	92.9%	85.7%	100%
5-year	71.8%	69.8%	63%	68.0%	61.5%	62.5%

Age of recipient values are given in years and expressed as mean and SD or median and interquartile ranges.

## Data Availability

Data of this study will be in the public archive of Charité-Universitätsmedizin Berlin, Germany.
